# Arterial Thoracic Outlet Syndrome in a Runner

**DOI:** 10.7759/cureus.15225

**Published:** 2021-05-25

**Authors:** Andrew J Ernst, Bryan Lamb, Christopher White

**Affiliations:** 1 Physical Medicine and Rehabilitation, Medical College of Wisconsin, Wauwatosa, USA

**Keywords:** arterial thoracic outlet syndrome, atos

## Abstract

Thoracic outlet syndrome (TOS) most commonly manifests in overhead athletes (e.g., baseball pitchers, swimmers, weight lifters) due to nerve compression caused by skeletal abnormalities. We present the case of a 43-year-old recreational runner with unilateral upper extremity pain while running. Vascular imaging identified an aberrant subclavian artery origin with positional compression in the absence of cervical bone anomalies confirming arterial TOS. A first rib resection and anterior scalenectomy led to symptom resolution. This case emphasizes the importance of a broad differential and complete workup in non-overhead athletes presenting with symptoms consistent with neurogenic TOS, as vascular interventions may be necessary to prevent future complications.

## Introduction

Thoracic outlet syndrome (TOS) is the clinical manifestation of neurovascular bundle compression at the thoracic outlet which most commonly presents in overhead athletes due to nerve compression. This case report describes a unique case of positional arterial thoracic outlet syndrome (aTOS) in a non-overhead athlete with aberrant vasculature and no cervical bone abnormalities.

## Case presentation

A 43-year-old female recreational runner without significant past medical history presented with a five-year history of intermittent, progressive right upper extremity pain while running. The pain began as diffuse cramping and pressure along her right arm and forearm present only while running. The pain would resolve shortly after the cessation of running. Over time, the symptoms progressed to include numbness and tingling in the ulnar forearm and dorsal hand as well as the first, fourth, and fifth digits. Symptoms increased in frequency and severity until they were noticeable with prolonged typing at work.

**Figure 1:**
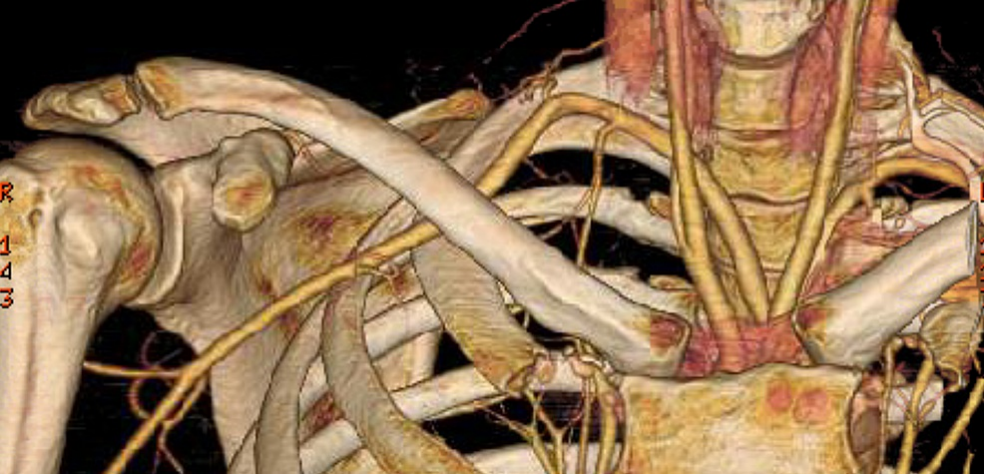
CTA with three-dimensional rendering in neutral shoulder position showing patent right subclavian artery. CTA: computed tomography angiography

**Figure 2:**
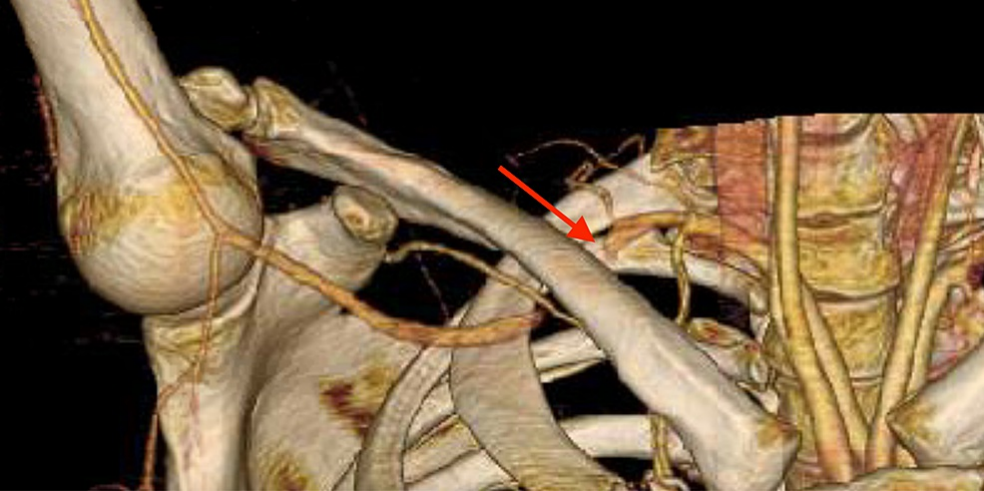
CTA with three-dimensional rendering in shoulder abduction showing extrinsic compression of the right subclavian artery. CTA: computed tomography angiography

Given the distribution of symptoms, differential diagnoses included TOS, peripheral neuropathy, and radiculopathy. Electromyography (EMG) showed evidence of mild right carpal tunnel syndrome and progressive right ulnar nerve entrapment across the elbow. Magnetic resonance imaging (MRI) of the cervical spine and non-contrast brachial plexus MRI revealed no acute pathology. Upon examination, the patient had 5/5 strength throughout bilateral upper extremities. Sensation to light touch and pinprick were intact for bilateral C3-T1 dermatomes. Tinel’s sign was negative at the elbow and wrist. Phalen and Spurling’s tests were also negative. With prolonged bilateral shoulder abduction, there was numbness in the right hand. Radial pulses were 2+ and symmetric. Blood pressures were 116/81 in the left brachial artery and 102/71 in the right brachial artery. Arterial studies of bilateral upper extremities revealed a significant decrease in digital pressures bilaterally with 180 degrees of shoulder abduction and exaggerated military positioning. Computed tomography arteriogram (CTA) revealed an aberrant origin of the right subclavian artery off the aortic arch. Without shoulder abduction, the right subclavian and axillary arteries were patent and normal in caliber (Figure 1). However, with shoulder abduction, there was a significant extrinsic compression of the right subclavian artery immediately distal to the interscalene triangle and along the entire course within the costoclavicular space (Figure 2) confirming the diagnosis of aTOS. Vascular surgery performed a partial right first rib resection with anterior scalenectomy for symptom relief and to decrease the risk of arterial degeneration from repetitive trauma. This was tolerated well without complications. At her two-week follow-up, the patient noted only mild numbness in her fourth and fifth digits after typing for multiple hours. There was no report of formal post-surgical rehabilitation. The patient has since been lost to follow-up.

## Discussion

TOS is the clinical manifestation of neurovascular bundle compression in either the scalene triangle, costoclavicular space, or pectoralis minor space [1,2]. This compression results in pain, paresthesias, weakness, and fatigability in the upper extremity. There are three distinct types of TOS: neurogenic TOS which results from brachial plexus compression, venous TOS which results from subclavian vein compression, and aTOS which results from subclavian artery compression [1-3]. Though aTOS accounts for only 1% of all TOS cases, it can lead to significant disability if not properly diagnosed and treated [1-3].

Classically, overhead athletes such as baseball pitchers, swimmers, and weightlifters represent a distinct patient population with a higher predisposition for developing TOS. The repetitive shoulder activities of these athletes can lead to scapular dyskinesia caused by hypertrophied scalene and pectoralis minor muscles which can result in compression of the neurovascular bundle at the thoracic outlet [1-3]. Other risk factors for aTOS include cervical ribs, first rib anomalies, fibrocartilaginous bands, and aberrant vasculature origins or courses [1-4]. It is possible for the etiology to be multifactorial, as was likely the case for the runner presented in this report. We hypothesize that this patient’s symptoms were a result of a thoracic outlet anomaly which was likely exacerbated by abnormal arm positioning while running. As a result of the repetitive trauma and compression of the subclavian artery, there is progressive intimal damage, stenosis, and post-stenotic dilation which contribute to fusiform aneurysms, mural thrombosis, distal embolization, and ischemia [1-4].

Though physical examination maneuvers have been used for the initial evaluation of TOS, the definitive diagnosis can only be made through vascular studies to include segmental Doppler, CTA, and magnetic resonance angiography [1]. Treatment is conservative for asymptomatic patients without evidence of arterial degeneration, but surgical intervention is necessary for those who are symptomatic or have arterial disease [1,3]. Non-surgical attempts to decompress the neurovascular bundle include scalene muscle stretching, botulism toxin injection into the scalene muscles, and diaphragmatic breathing techniques to decrease the workload of the scalenes during intense aerobic exercise [5-7]. Maintenance of good posture during activity as well as avoidance of overhead arm positioning while sleeping may also help with neurovascular compression [8]. Failure of these interventions may necessitate the avoidance of overhead activities that cause symptoms. Surgical interventions for aTOS begin with the removal of anomalous ribs or fibrous bands [1]. Subclavian artery repair is indicated for arterial degeneration, dilation, or aneurysm to prevent upper limb ischemia [9]. After surgery, patients should commit to a rehabilitation program that addresses muscle imbalances (e.g., weak upper trapezius, levator scapulae, and sternocleidomastoid relative to hypertrophied scalenes, lower trapezius, and pectoralis) and range of motion exercises [10]. Strengths of this case report include an extensive workup, including physical examination and imaging studies, clear imaging to support diagnosis, and satisfactory response to treatment. The major limitation is the lack of documented post-surgical rehabilitation as well as long-term follow-up.

## Conclusions

In conclusion, aTOS is the clinical manifestation of vascular compression associated with pain, paresthesias, weakness, and fatigability of the ipsilateral upper extremity. Vascular imaging confirms the diagnosis, and definitive treatment includes the surgical resection of structures causing vascular compression. Though aTOS most frequently presents in overhead athletes, it should remain on the differential for any patient with the symptom cluster described previously. Early intervention prevents vascular insufficiency and distal ischemia.

## References

[1] Hussain MA, Aljabri B, Al-Omran M (2016). Vascular thoracic outlet syndrome. Semin Thorac Cardiovasc Surg.

[2] Vemuri C, McLaughlin LN, Abuirqeba AA, Thompson RW (2017). Clinical presentation and management of arterial thoracic outlet syndrome. J Vasc Surg.

[3] Duwayri YM, Emery VB, Driskill MR, Earley JA, Wright RW, Paletta GA Jr, Thompson RW (2011). Positional compression of the axillary artery causing upper extremity thrombosis and embolism in the elite overhead throwing athlete. J Vasc Surg.

[4] Criado E, Berguer R, Greenfield L (2010). The spectrum of arterial compression at the thoracic outlet. J Vasc Surg.

[5] Danielson K, Odderson IR (2008). Botulinum toxin type A improves blood flow in vascular thoracic outlet syndrome. Am J Phys Med Rehabil.

[6] Novak CB (2003). Thoracic outlet syndrome. Clin Plast Surg.

[7] Brismée JM, Phelps V, Sizer P (2005). Differential diagnosis and treatment of chronic neck and upper trapezius pain and upper extremity paresthesia: a case study involving the management of an elevated first rib and uncovertebral joint dysfunction. J Man Manip Ther.

[8] Crosby CA, Wehbé MA (2004). Conservative treatment for thoracic outlet syndrome. Hand Clin.

[9] Patton GM (2004). Arterial thoracic outlet syndrome. Hand Clin.

[10] Wishchuk JR, Dougherty CR (2004). Therapy after thoracic outlet release. Hand Clin.

